# The genesis of OH-stretching vibrational circular dichroism in chiral molecular crystals†

**DOI:** 10.1039/d4sc08055f

**Published:** 2025-04-22

**Authors:** Sascha Jähnigen, Rodolphe Vuilleumier, Anne Zehnacker

**Affiliations:** a Department of Biology, Chemistry, Pharmacy, Freie Universität Berlin 14195 Berlin Germany sascha.jaehnigen@fu-berlin.de +49 30 838 54568; b Chimie Physique et Chimie du Vivant, Département de Chimie, Ecole Normale Supérieure, PSL University, Sorbonne Université, CNRS 75005 Paris France; c Institut des Sciences Moléculaires d’Orsay (ISMO), CNRS, Université Paris-Saclay 91405 Orsay France

## Abstract

The stretching vibration of hydroxyl groups, *ν*(OH), appears with a strong absorption in the 3 μm region of the infrared (IR) spectrum. In chiral molecular crystals, also vibrational circular dichroism (VCD) can be observed for this band, which is demonstrated by the example of two chiral alcohols crystallising with space groups *P*2_1_ and *P*3_1_21, respectively. Measurements demonstrate that the VCD bands of the *ν*(OH) mode show an increased fine structure in comparison to the broad infrared absorption bands. In a computational study, the chiroptical signal can entirely be traced back to non-local terms emerging from the supramolecular environment, determined by the hydrogen-bonded network involving the hydroxyl groups. In turn, the VCD of individual molecules in the crystal related to the *ν*(OH) mode is almost zero. It can thus be concluded that the entire VCD band in the 3 μm region is determined by the chirality of the crystal, but not by that of the molecules. Further analysis reveals that while vibrational coupling mainly arises from the hydrogen-bonded network, the VCD is strongly influenced by the weaker interactions and long-range order. This highlights the significance of the OH stretching mode as a sensitive probe of supramolecular chirality.

## Abstract 2

The VCD signal observed in a crystal for the stretching vibration of hydroxyl groups is entirely determined by the crystal symmetry, whereas it vanishes at the molecular level.

## Graphical abstract

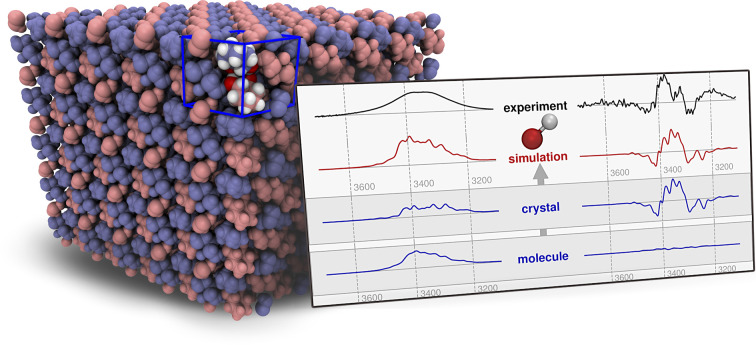

## Introduction

The characterisation of molecular substances in the solid state is of paramount importance in materials science, drug discovery and catalysis.^1,2^ While crystal structure resolution constitutes an essential part of the characterisation of newly synthesised molecules, the preparation of single crystals (*e.g.*, for X-ray diffraction measurements) can be cumbersome. In situations where the atomic structure remains unidentified, spectroscopic techniques can be utilised to retrieve missing information through the sample's interaction with light. Vibrational spectroscopy employs radiation in the mid-infrared (mid-IR) region, offering a versatile and cost-effective approach to investigating molecules in the solid state. Valuable information about the molecules and their non-covalent network is provided by the characteristic frequency and intensity of vibrational modes localised in the functional groups. Mid-IR absorption spectroscopy has become a routine technique for probing vibrational transitions, particularly in the fingerprint region (*i.e.* below 2000 cm^−1^).^3^

Vibrational circular dichroism (VCD) is a measure of the vibrational optical activity of a sample, defined as the differential absorption of left-handed and right-handed circularly polarised light in the mid-IR region.^4,5^ VCD is highly sensitive to conformational and environment effects because it combines two important components: its magnitude depends, on the one hand, on the simultaneous change in the electric and magnetic dipole moments, including their relative angle, which is related to the Rosenfeld equation of circular dichroism.^6^ On the other hand, it probes the optical activity associated with the vibrational modes, addressing both molecular and supramolecular structures and showing a certain degree of delocalisation.^7^ VCD thus allows conclusions to be drawn about molecular and supramolecular geometry that go far beyond the capabilities of conventional IR absorption spectroscopy or other chiroptical techniques.^8^ A number of studies have reported on how VCD can be used to investigate solvation effects,^9–12^ hydrogen bonding,^13^ the structure of ionic liquids,^14,15^ the competition between inter- and intramolecular interactions,^16–19^ or cluster formation^11,20–22^ and finds promising applications in natural product analysis^23,24^ and catalysis.^25^

Solid-state measurements of VCD have gained attention in recent years due to improved experimental setups and advances regarding solid-state calculations that are required for their interpretation.^26–33^ Crystalline systems represent a distinctive category of VCD applications, characterised by the ubiquitous presence of geometrically stable non-covalent interactions between molecules, which leads to a strong delocalisation of the intramolecular vibrational modes. Indeed, the structure of solid samples is characterised by frozen molecular conformations and in many cases by the presence of chirality at both the molecular and supramolecular levels, giving rise to non-local VCD terms.^7^ It has been shown, that supramolecular connections, such as hydrogen bonds (HB), but also weaker interactions, have a significant influence on a solid-state VCD spectrum, which holds great potential for pharmaceutical applications.^34–39^

The 3 μm region of IR and VCD spectra (*i.e.*, >3300 cm^−1^) holds particular potential for the study of organic crystals.^40^ While the fingerprint region collects an overwhelming multitude of narrow bands, that region is solely dominated by the stretching vibration of the OH-group, *ν*(OH), which is usually engaged in the HB network of the crystal. Consequently, organic compounds with carboxylic, hydroxyl or amide functions absorb strongly at characteristic positions above 3300 cm^−1^. In the case of IR absorption, significant broadening of the peaks renders the spectrum less informative and more difficult to interpret. VCD, on the other hand, has the potential to resolve this region because it is more sensitive to structural effects and supramolecular geometries. The primary challenge in interpreting and theoretically predicting solid-state VCD lies in establishing a link between the crystal structure, the vibrational couplings, and the observed spectra, using appropriate reference calculations.

Generally, quantum mechanical calculations are used to obtain, on the one hand, the vibrational absorption frequencies and normal modes and, on the other hand, the dipolar response of the electronic system to the vibrational motions of the atoms.^4,41^ In the case of solid state samples, the model system must not only reflect the geometry of the molecular stack, but also include the mid- to long-range vibrational couplings, leading to models of considerable size. Although Kohn–Sham density functional theory (KS-DFT) can provide sufficient accuracy, the computational cost of a VCD calculation scales unfavourably with system size. To reduce this cost, methods based on smaller clusters extracted from the crystal structure have been proposed.^36,37,42–45^ Furthermore, previous studies have demonstrated that fragmentation approaches in combination with a tensor transfer can yield satisfactory results while maintaining an acceptable computational cost.^27,46,47^

Recently, we introduced the formalism for performing VCD calculations under periodic boundary conditions, allowing for full-scale crystal models based on unit or supercells.^26^ This approach relies on conventional solid-state KS-DFT calculations using a plane-wave basis for the electronic structure and periodic nuclear velocity perturbation theory (NVPT) to obtain the electronic response.^48–50^ By decomposing the system into local subsystems (*e.g.*, molecules embedded in the crystal), followed by their recombination, non-local VCD terms emerge as cross-terms between different units, which is related to earlier fragmentation concepts and the coupled oscillator model.^51–53^ In a series of applications, this methodology has been combined with *ab initio* molecular dynamics (AIMD) simulations, which yield highly accurate vibrational dynamics without the inherent approximations of the static picture that require minimised geometries and the calculation of a Hessian matrix.^26,41,54–56^ Through a subspectra analysis, a link between crystal chirality and non-local VCD could be established.^56^

**Fig. 1:**
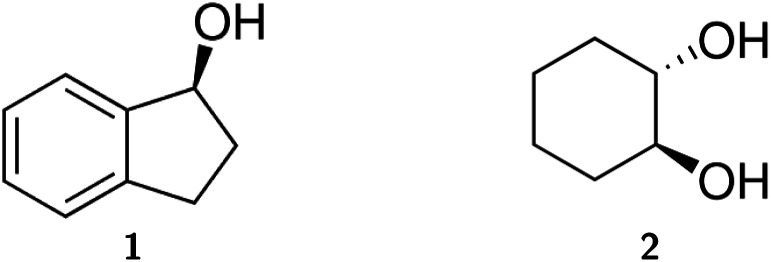
Lewis formulae of (*S*)-(+)-1-indanol (1) and (1*S*,2*S*)-*trans*-1,2-cyclohexanediol (2).

In this study, we present an experimental and theoretical investigation utilising IR and VCD spectroscopy in the *ν*(OH) region to examine molecular crystals of (*S*)-(+)-1-indanol (1) and (1*S*,2*S*)-*trans*-1,2-cyclohexanediol (2) (Fig. 1). While compound 1 crystallises in the non-chiral Sohncke space group *P*2_1_, where the chirality of the crystals is determined by the chirality of the asymmetric unit, crystals of 2 belong to the chiral Sohncke space group *P*3_1_21, which contains a (chiral) threefold screw axis.^7^ In previous reports on both systems, we presented the fingerprint spectra, composed of both local and non-local terms, as a representation of the multi-scale chirality found in the crystals.^26,56^ This study demonstrates that, at the single molecular level, the vibration of the OH group, *ν*(OH), does not show VCD. However, it does exhibit strong non-local terms that originate from the crystal structure. These terms are closely associated with the symmetry operations inherent to the unit cell. The isolation of crystal-induced effects on the VCD spectrum represents a promising avenue for unambiguously investigating polymorphism, particularly in the context of pharmaceutical applications.^32^ In addition, VCD has a greater reach into space than IR absorption, which enhances its sensitivity to supramolecular chirality.

## Theory

VCD denotes the difference in the absorption of left- and right-circularly polarised light, Δ*α* = *α*_L_ − *α*_R_ in the mid-IR region of the electromagnetic spectrum.^4^ For a given vibrational transition *a*, the sign and magnitude of a VCD signal depend on the rotational strength, *R*_*a*_, defined through the Rosenfeld equation,^6,57^

1 Δ*α* → *R*_*a*_ = Im(***μ***_*a*_·***m***_*a*_) = *ω*_*a*_^−1^Re(***j***_*a*_·***m***_*a*_)

with the electric transition dipole moment ***μ***_*a*_, the magnetic transition dipole moment ***m***_*a*_, the electric current dipole moment ***j***_*a*_ = d***μ***_*a*_/d*t*, and the angular frequency of the vibrational mode, *ω*_*a*_. The last term in eqn (1) represents the velocity formulation of the rotational strength, used in this study. Invoking the dynamic picture for vibrational dynamics, the rotational strength can be formulated in the Heisenberg representation as the Fourier-transformed time-correlation function (TCF) of the instantaneous dipole moments.^26,49,54,58,59^ In the classical limit, the latter can be obtained from an MD trajectory, whereby the expression for the rotational strength becomes^60,61^2
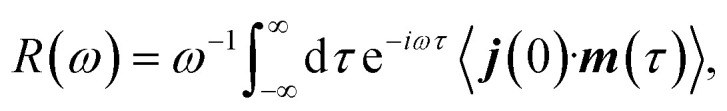
with the lag time or correlation depth *τ*. Solving eqn (2) does not require optimised geometries or a harmonic frequency analysis of the target molecules—common approximations in static calculations—which makes this approach suitable for complex systems in the bulk phase and periodic boundaries.^41^ It depends, however, on an accurate description of the potential energy surface in order to yield reliable vibrational dynamics. This can be achieved through AIMD simulations, where the energies and forces are derived directly from the electronic wave function.^62^

While in principle the observables entering eqn (1) or (2) can be formulated within the frameworks of KS-DFT, the magnetic dipole moment ***m***(*t*), representing a dynamic property of the electronic structure, depends on additional terms beyond the Born–Oppenheimer (BO) approximation.^4,63^ In the context of MD-based VCD calculations, these terms can be added by means of quantum linear response theory, specifically using nuclear velocity perturbation theory (NVPT). The BO-Hamiltonian 
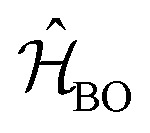
 is augmented with an adiabatic imaginary coupling term, derived from the electron-nuclear coupling operator of the Exact Factorisation.^48,63,64^ Hence, the modified electronic structure problem to be solved reads3
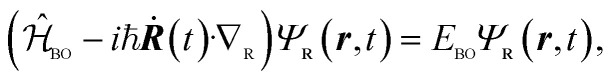
with nuclear velocities ***Ṙ***(*t*), obtained from the MD trajectory; the gradient with respect to the nuclear coordinates ∇_R_; and the reduced Planck constant ℏ. *Ψ*_**R**_(***r***, *t*) is the complete adiabatic wavefunction that comprise the BO ground state and an imaginary first-order correction. *E*_BO_ denotes the BO potential energy surface, which remains unaffected by the correction. This allows for AIMD simulations to be conducted within the BO approximation in order to derive the vibrational density of states. Eqn (3) can then be solved through density functional perturbation theory (DFPT) taking the (small) nuclear velocities as perturbation parameter.^48,50^ The magnetic dipole moment is obtained from the cross product of the position and the momentum operator,^4^4
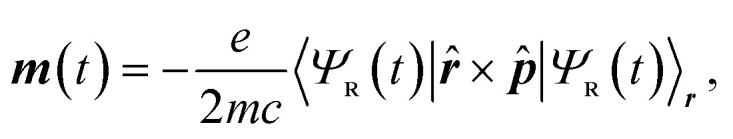
with elementary charge *e*, electron mass *m*, and speed of light *c*.

The NVPT formalism is particularly useful when working with periodic boundary conditions, as it allows plane-wave based calculations in the solid state, thus avoiding the need to deal with the velocity gauge in the atom-centred basis functions of the electrons. Moreover, the exact decomposition of the dipolar response can be achieved through the definition of localised electronic orbitals, such as Wannier functions.^49,65^ By applying such a fragmentation approach to eqn (2), the dipolar responses can be defined as the sums, ∑_*k*_ and ∑_*l*_, over local contributions of individual units (*e.g.*, molecules). This leads to a decomposition of the TCF into local and non-local terms,5
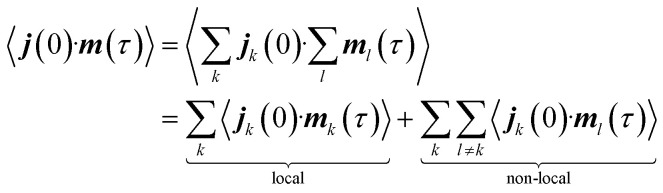


The magnetic dipole moment is a pseudovector and its magnitude thus depends on the relative position with respect to a chosen origin, 
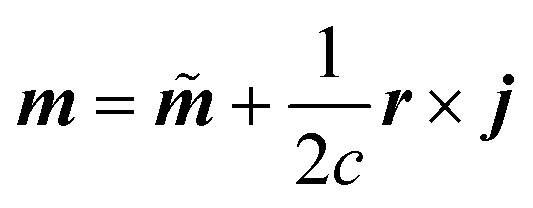
. Introducing this gauge dependence into the TCF splits the non-local into a direct coupling (DC) and an origin-dependent gauge transport (GT) term (eqn (S1)†).^7,56^ Both terms have been shown to be equally important for the simulation of solid-state VCD.^56^ Under periodic boundary conditions required for models of molecular crystals, the GT term takes a slightly different form, as shown in eqn (S2),† to avoid spurious terms due to superfluous lattice translations.^26^

Analogous expressions can be found for the local and non-local contributions to the vDOS and IR absorption (*cf.* eqn (S3) and (S4)†), but in this case, no gauge transport term scaling with the distance occurs.

## Methodology

The VCD spectra were recorded on commercially available samples of 1 and 2 dispersed in a KBr pellet, using a Fourier Transform Infra-Red (FTIR) spectrometer Vertex 70 equipped with a VCD module PMA 50 (Bruker), and following the previously described procedure.^26,36,37,56,66–70^ Special care has been taken to prepare an isotropic sample so that the Rosenfeld equation is valid and to minimise linear birefringence and linear dichroism, as described in the procedure outlined in the ESI.† The mirror image relationship between the two enantiomers has been verified and is shown in Fig. S1.†

The computational study is based on AIMD simulations with the CP2K software package^71–73^ using the B3LYP functional^74–78^ with Grimme's dispersion correction (D3),^79^ GTH pseudopotentials,^80–82^ the DVZP-MOLOPT-SR basis set^83^ with a density cutoff of 400 Ry, and the Auxiliary Density Matrix Method (ADMM) for Hartree–Fock Exchange Calculations using the cFIT3 auxiliary basis^84^ and a cutoff radius of 4.95 Å.^85^

The electronic response for the computation of VCD was obtained with projected periodic NVPT calculations using the BLYP functional,^75,76^ MT pseudopotentials,^86,87^ and a plane wave cutoff of 100 Ry based on our own development version of the CPMD code, available on GitHub.^26,48,49,88^

The local response observables were obtained based on maximally localised Wannier functions (MLWFs) that were calculated from the electronic structure.^65^ In this procedure, the Wannier centres are mapped to their corresponding molecular centre of mass *via* their nearest heavy atom, and their magnetic gauge is shifted accordingly.^49^ This defines the units used in the fragmentation approach in eqn (5). Further details of the simulation procedure can be found elsewhere for compounds 1 (ref. 56) and 2 (ref. 26).

The computed spectra have been scaled in the frequency domain by a factor of 0.95 and 0.97 for compounds 1 and 2, respectively. It should be noted that in this AIMD setup the nuclei are treated as point-like particles and nuclear quantum effects (NQEs) are not considered. While there are schemes to improve vibrational spectra with NQEs,^89,90^ we do not observe a significant impact on the VCD result of the systems considered. However, we do not believe that this holds for all types of systems, and the effect of NQEs in the 3 μm region should definitely be further investigated for the solid state.^41^

All post-processing, the evaluation of the periodic gauge, the calculation of the Fourier-transformed TCF, as well as the visualisation was carried out with our python library ChirPy available on GitHub.^91^

In order to perform an angular analysis of the dipole moments, all oscillations bound to the 3 μm region were extracted from the MD trajectory using a Fourier filter in the limits of 3100 and 3450 cm^−1^. The group correlations were calculated based on the non-local contributions of all molecular pairs *ab* as the integrated absolute spectra within *ω*_u_ = 3100 cm^−1^ and *ω*_o_ = 3600 cm^−1^, normalised with respect to the local contributions. In order to obtain the non-local correlation strength *C*_*A*_ of the symmetry operation *A*, the average is taken over all molecular pairs *ab* that are related by *A* (*cf.* Fig. S3 and eqn (S5)†),6
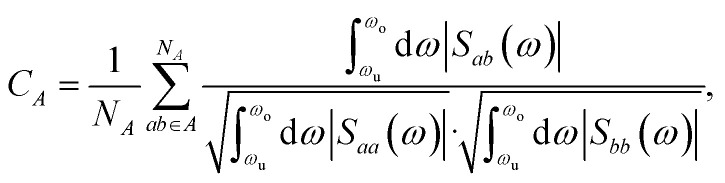
with *S*_*ab*_(*ω*) being the spectral cross section (*i.e.*, vDOS, IR absorption, or VCD) between molecules *a* and *b*; and *N*_*A*_ being the number of pairs *ab* involved in symmetry class *A*.

The radially resolved plots were obtained by means of a regularisation procedure described previously:^55,92^ The TCF of two vectorial signals ***a***(*t*) and ***b***(*t*), 〈***a***(0)·***b***(*τ*)〉, is described in terms of a spatially-resolved TCF, 〈***a***(0, 0)·***b***(*τ*, *r*)〉,7
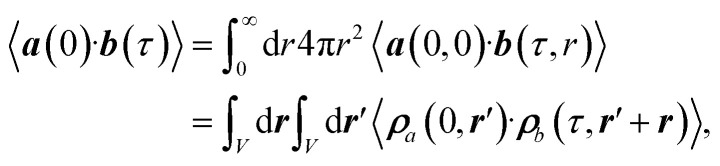
with radius *r*, volume integral 
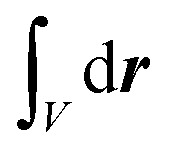
, and local densities ***ρ***_*a*_(*t*, ***r***) and ***ρ***_*b*_(*t*, ***r***). In this work, the local dipole and velocity densities were smoothened with a regularisation parameter of *σ* = 0.4 Å and mapped on a three-dimensional Cartesian grid using 18 equally-spaced grid points for each dimension.

The raw data as well as detailed implementations for reproducing all the results shown can be found in the supplementary data on Zenodo/GitHub.

## Results and discussion

**Fig. 2:**
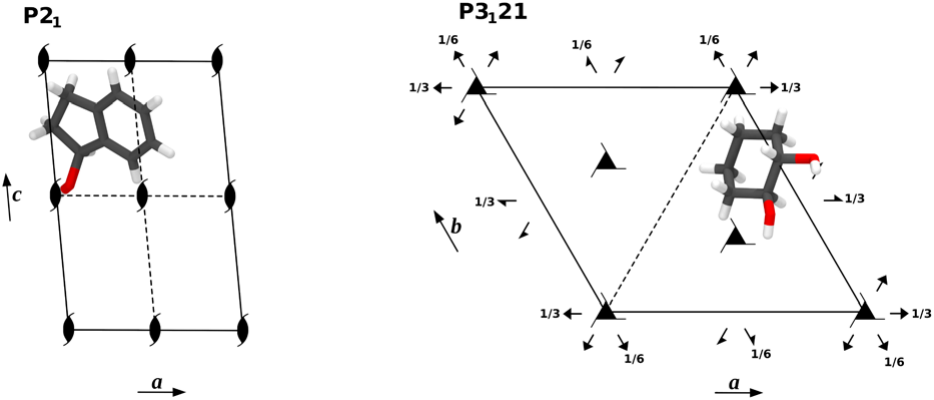
Space group diagrams of compounds 1 (left) and 2 (right) with asymmetric unit.

Crystals of compound 1 are monoclinic and contain a single twofold rotary-translation (screw axis) as non-trivial symmetry operation to construct the unit cell from the asymmetric unit, the latter being a single molecule (Fig. 2, left).^56^ The molecules are held together by a variety of non-covalent interactions, most notably by hydrogen bonds (HBs) connecting the OH-groups. This suggests that these linker groups are more sensitive to changes in the supramolecular environment than the less exposed parts of the molecule.

**Fig. 3:**
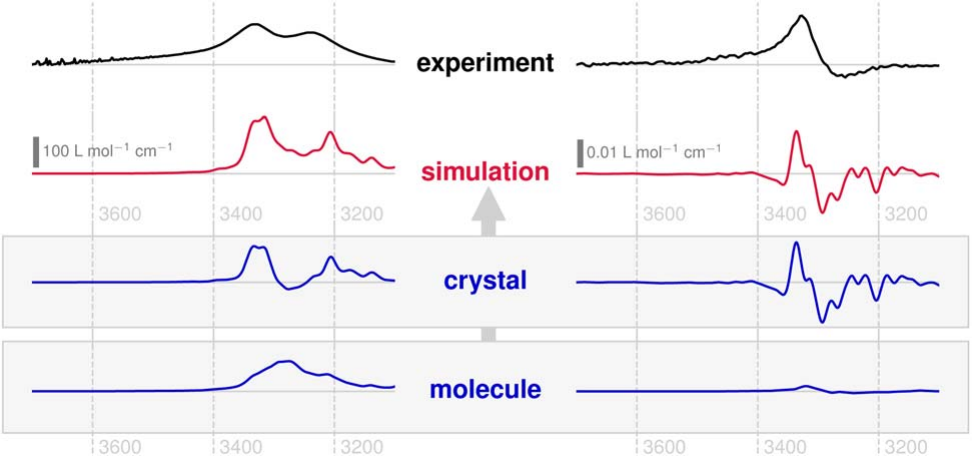
Solid-state IR absorption spectra (left) and VCD spectrum (right) of compound 1, obtained from KBr pellets (black) as well as predicted from periodic AIMD-NVPT calculations (red). The experimental spectra have been scaled to approximately match the intensities. The lower shaded panel shows the calculated subspectra that make up the simulated spectrum corresponding to molecular and crystal chirality (blue).

Fig. 3 depicts the experimental and the theoretical IR and VCD spectra, obtained in the 3 μm region, of compound 1 in the solid state. In the IR measurements the expected broad *ν*(OH) absorption band can be found as distinct double peaks, together with a corresponding bisignate VCD band. This points to an in-phase/out-of-phase coupling of neighbouring oscillators (*i.e.*, HB-linked OH groups). The simulated spectra, based on AIMD and periodic NVPT calculations, reproduce the experimental data very well. In analogy to previous work, the VCD response can be partitioned into local and non-local terms,^7^ so as to obtain the subspectra at the molecular and crystalline scales, respectively.

Indeed, the simulations support the conclusion that a coupled oscillator setup dominates the IR and VCD spectra of compound 1, since the corresponding double/bisignate peaks only appear at the crystalline scale and are not located on individual molecules. As a remarkable result, however, the molecular VCD for this band is almost zero. This stands in contrast to the fingerprint region, where a multitude of local peaks emerge alongside non-local terms.^56^ Also for IR absorption, about 50% of the band is found locally as a structureless signal.

**Fig. 4:**
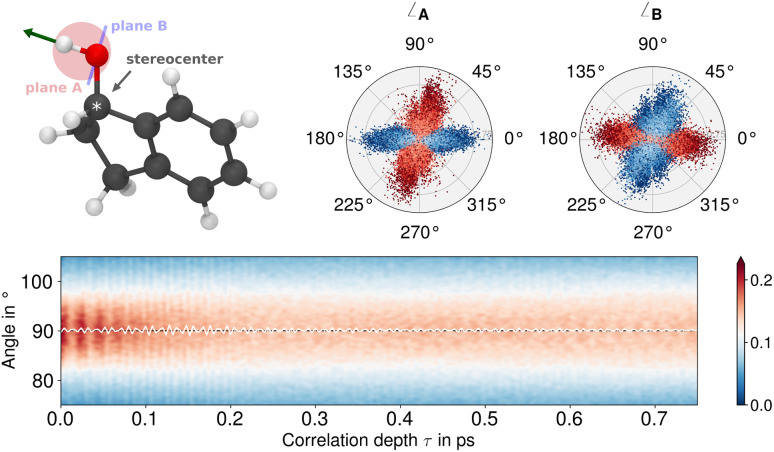
Geometry of the molecular polarisation bound to the 3 μm region where the *ν*(OH) mode appears. (Upper left) Ball and stick model of compound 1 with mode vector (green) and two geometric planes A and B (see main text). (Upper right) Distribution of the angle between these reference planes and the electric current (blue) and magnetic dipole (red) moment vectors, respectively, obtained from the AIMD-NVPT simulations.^*a*^ (Bottom) Time correlation function of the relative angle distribution between the electric current and magnetic dipole moment bound to the 3 μm region together with the corresponding mean value (white). The colours corresponds to the relative probability (*cf.* colour bar to the right), whereas the *y*-axis integrates to one. ^*a*^Normalisation: the unit circle corresponds to 213.92 Debye per ps (current dipole) and 144.244 Debye-Å per ps (magnetic dipole).

Fundamentally, the transition dipole moment bound to the *ν*(OH) vibration, determined by the inherent (changing) polarity of the OH bond, does not vanish on a local scale and yields a detectable IR signal. However, for the definition of VCD, the magnitudes of the transition dipole moments (electric and magnetic) alone are not sufficient. In accordance with the Rosenfeld equation (eqn (1)), the rotational strength is dependent upon the scalar product of the two vectors, which introduces their relative angle as an additional determinant. This well-established chiroptical principle implies that the (V)CD signal vanishes for perpendicular transition dipole moments, irrespective of their magnitude. Fig. 4 depicts the local dipolar fluctuations linked to the *ν*(OH) vibration of individual molecules as embedded in their crystal structure, extracted from the AIMD trajectories of compound 1. The angular distribution of the electric (current) dipole (blue) and magnetic dipole moment (red) is illustrated with respect to pertinent planes established on the molecular geometry: One defined by the COH moiety (plane A), another takes the OH bond axis as its normal vector (plane B) (*cf.*Fig. 4, top left). In both cases, either the electric or magnetic dipole moments assume a largely parallel alignment with one plane, respectively, while their counterparts oscillate with almost orthogonal geometry. The moment's relative orientation remains perpendicular even as the time-correlation function's lag time increases (Fig. 4, bottom), so that no constructive “Rosenfeld angle” is ever obtained. The only correlation effect found is a periodic broadening and narrowing of its distribution with a 23 fs period, which is due to the wagging of the OH group at about 1450 cm^−1^ in the fingerprint region.^56^ However, this coupling with fingerprint vibrational modes has no effect on the average angle between the moments in the *ν*(OH) region.

Combining the information provided, it can be concluded that the electric and magnetic dipole moments of the individual molecules in 1 almost align with the geometry of the OH group. They largely maintain their orthogonal orientation, which explains that the *ν*(OH) vibration is almost optically inactive on a molecular scale. This is an interesting observation, given that the OH group directly binds to the chiral centre of the molecule (Fig. 4, top left), apparently without significant effect on the strongly localized bond polarization. It will be shown in the following that chirality has an impact when moving to the supramolecular scale.

At the level of the crystal, the oscillating dipole moments are coupled due to the delocalisation of the vibrations and in accordance with the supramolecular geometry. They produce non-local VCD terms, either through direct coupling (DC) or gauge transport (GT), as previously described.^56^ In the case of the compound 1, it is found that the entire non-local VCD signal of the *ν*(OH) band emerges from the 2_1_ symmetry operation *via* the GT term (*cf.* Fig. S4†). As this term is defined solely in terms of the changing electric dipole moments and their relative position, this finding is tantamount to assuming a coupled oscillator mechanism behind the observed VCD signal.

Compound 2 crystallises in the chiral Sohncke space group *P*3_1_21, which possesses one enantiomorphic symmetry operation *via* a threefold screw axis.^26^ Furthermore, the group contains a twofold rotation axis that combines with the screw axis, rendering a set of six different symmetry relations, including the identity (Fig. 2, right and Table S1†). The asymmetric unit consists in one chiral molecule and the obtained crystals are trigonal. Given that compound 2 carries two OH groups, the resulting three-dimensional crystal structure is considerably more complex than that of compound 1. The intertwined HB scaffold is sustained by the OH groups, which makes them susceptible to supramolecular vibrational effects.

**Fig. 5:**
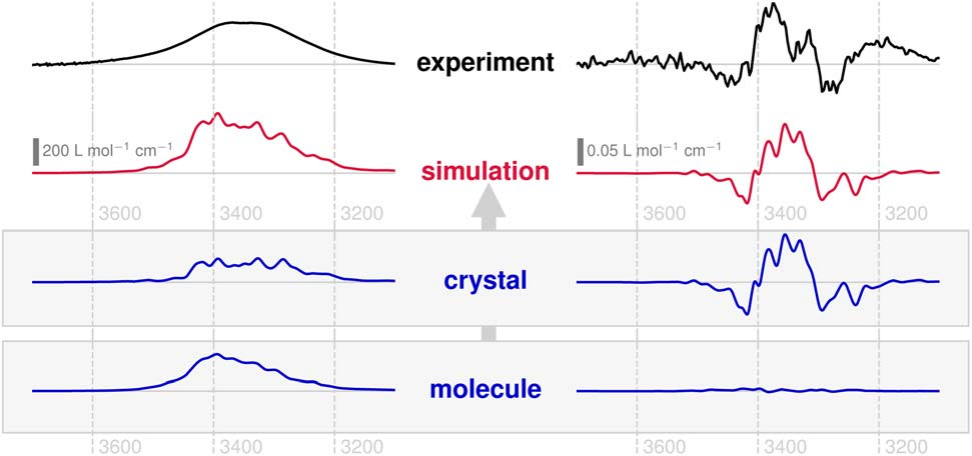
Solid-state IR absorption spectra (left) and VCD spectrum (right) of compound 2, obtained from KBr pellets (black) as well as predicted from periodic AIMD-NVPT calculations (red). The experimental spectra have been scaled to approximately match the intensities. The lower shaded panel shows the calculated subspectra that make up the simulated spectrum corresponding to molecular and crystal chirality (blue).

The experimental and theoretical IR and VCD spectra are shown in Fig. 5. The absorption band is notable for its lack of structure, in contrast to the chiroptical band, which displays a distinctive −+− pattern. Both the broad absorption band and the delicate VCD shape of the *ν*(OH) vibration hint to a much more complex coupling mechanism than that found for compound 1.

The simulated spectra based on AIMD and periodic NVPT calculations show very good agreement with the measurements. Although the IR absorption band contains considerable non-local contributions (about 30% of the total intensity), these do not influence its overall shape and therefore do not convey any information about the crystal structure. As in the case of compound 1, no VCD can be found at the molecular level of compound 2, but the entire band is carried by the crystal structure. Even the fact that compound 2 carries two OH groups per molecule (both directly bound to the chiral centres, respectively) does not yield local VCD, as these groups are not hydrogen-bonded to each other and therefore vibrationally decoupled (*cf.* Fig. S3,† right). The VCD associated with the *ν*(OH) vibration in compounds 1 and 2 seems to follow similar patterns, exhibiting localised and perpendicular dipolar responses on a molecular scale, whose coupling is driven by delocalised vibrational motion.

In order to assess for compound 2 the influence of crystal packing, it is convenient to classify the non-local terms contributing to the vibrational density of states (vDOS), IR absorption, and VCD according to the space group symmetry (eqn (S5)†). Fig. S2† illustrates the symmetry relations found between molecular pairs (*cf.* Table S1†). Next to the principal 3_1_ and 2 symmetry operations (shown in red and blue, respectively), there are mixed operations that result from the combinations 2 ⊕ 3_1_ and 2 ⊖ 3_1_ (shown in green). While the molecular clusters with 2 and 2 ⊖ 3_1_ symmetry are hydrogen bonded, either as dimer or as a chain, the pairs with 3_1_ and 2 ⊕ 3_1_ symmetry are not.

**Fig. 6:**
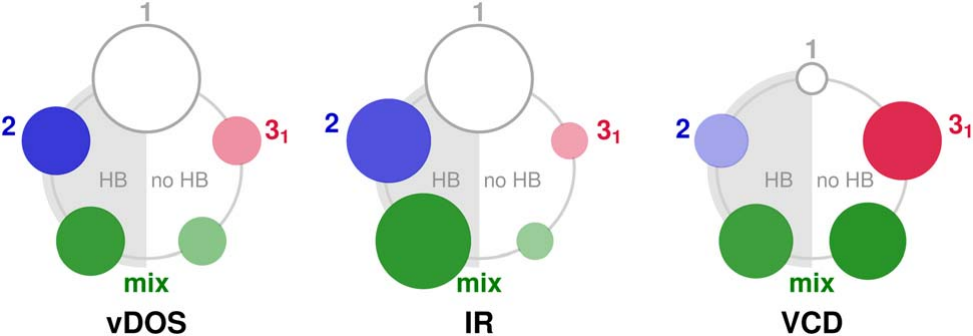
Representation of the non-local terms contributing to the predicted vibrational density of states (vDOS), the infrared absorption (IR), and the vibrational circular dichroism (VCD) of the *ν*(OH) mode in compound 2, resolved as pair couplings between molecules related by the same symmetry operation (*cf.* Table S1†). The area of the circles corresponds to the individual correlation strength that has been normalised with respect to the local contribution (empty circle) according to eqn (6).

Fig. 6 illustrates the distribution of vDOS, IR and VCD in the 3 μm region over these symmetry classes including the identity referring to the local term (“1” in Hermann Mauguin notation). The cross terms of the vDOS serve as a measure for the delocalisation of vibrational modes, which spans the entire unit cell with contributions observed in every symmetry class (Fig. 6, left). Up to 40% of the total vDOS is delocalised (see Fig. S3,† left, for a detailed correlogram), whereas the hydrogen bonded connections exhibit a higher ratio than those without hydrogen bonds. This result represents what can be expected from the packed and rigid crystal structure. It is important to note, however, the specific role of the hydrogen bonds communicating between individual *ν*(OH) oscillators of different molecular subunits, which ultimately contributes to the enhanced delocalization ratio.

The delocalised vibrational modes translate into non-local spectroscopic terms through the associated oscillator and rotational strength, respectively.^55^ In the case of the IR absorption (Fig. 6, middle), a pattern comparable to that of the vDOS emerges, yet the hydrogen bonded connections appear further enhanced with higher oscillator strength. This suggests an enhanced electric transition dipole moment associated with charge transfer between aligned local dipoles. Considering hydrogen bonding as a special type of donor–acceptor interaction, such an enhancement can be expected. For the other symmetry groups without hydrogen bonding, no such mechanism exists, resulting in a much lower oscillator strength.

The situation is markedly different in the case of VCD (Fig. 6, right). All symmetry classes show significant values for the rotational strength. The largest values are found for geometries that do not include a hydrogen bond, in particular the 3_1_ class, whereas 2 contributes the least to the non-local signal. This means, first, that non-local VCD does not have to strictly follow the delocalisation pattern of the vDOS and, second, that the supramolecular geometry has gained importance over the presence of hydrogen bonds. Indeed, the 3_1_ class with the lowest degree of delocalisation (around 20%) shows the strongest non-local VCD (about 7.5 times the local term), followed by the mixed classes that contain the 3_1_ operation as well. Given that the threefold screw axis represents an enantiomorphic symmetry operation that explicitly introduces “space group chirality” into the crystal, this may be regarded as an expected outcome. However, it is a noteworthy finding that VCD is capable of converting weak vibrational coupling into large chiroptical signals, as long as a stable geometry is maintained. The distinctive −+− VCD pattern is a consequence of the combination of GT and DC terms that constitute the band, reflecting the complex three-dimensional geometry of the unit cell (*cf.* Fig. S5†). Therefore, a simple coupled oscillator mechanism as for compound 1 cannot be invoked for compound 2.

**Fig. 7:**
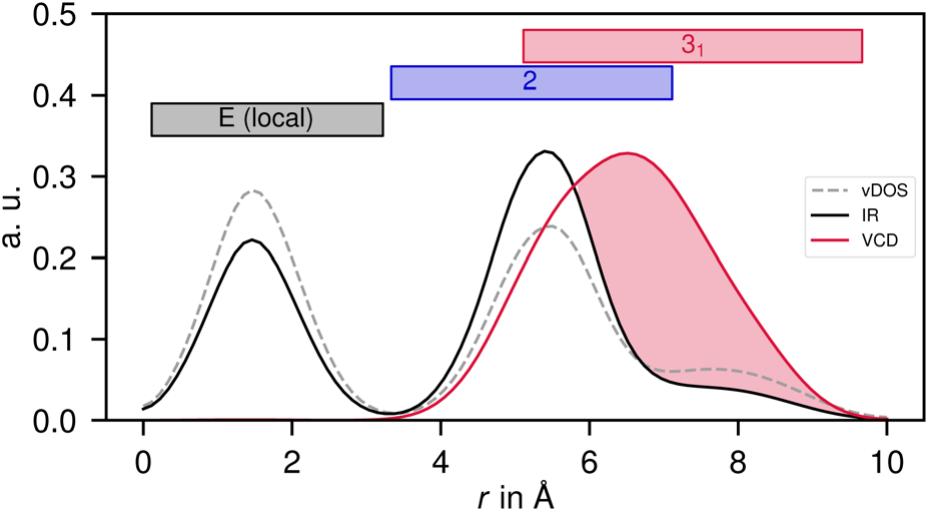
Radial resolution of the vibrational density of states (vDOS), the infrared absorption (IR), and the vibrational circular dichroism (VCD) of the *ν*(OH) mode in compound 2 with respect to the molecular centres of mass.

The symmetry operations of the *P*3_1_21 space group cover different spatial regions. Consequently, the non-local spectroscopic terms extend into space depending on the symmetry operation to which they are susceptible. Fig. 7 depicts the radial distribution of vDOS, IR and VCD in the 3 μm region of compound 2, compared to the geometric spatial range of the different symmetry operations. Since VCD is strong for all molecular cross couplings involving 3_1_ symmetry, it extends considerably further into space than the main contributions from non-local IR absorption or the delocalisation rate of the vDOS. This property allows VCD to “see” more deeply into space, thereby demonstrating its high relevance for solid state structure determination, particularly with respect to polymorphism with similar short-range but altered long-range order.^93,94^ The spanned radial range in Fig. 7 extends to the boundaries of the simulation cell, so that it cannot be ruled out that VCD sees even further. It is important to note, however, that VCD is fundamentally dependent on the presence of delocalised vibrational modes in the far-reaching regions as well. Fig. 7 is thus an ideal demonstration of how VCD can grow even on weak vibrational couplings.

The term “enhancement” is often used to describe the increase in signal typically observed upon crystal formation. The data available for compound 1 enable a comparison of the VCD spectra across different environments and spectral regions. In the fingerprint region, the local VCD signal makes a significant contribution to the total VCD signal in the solid state. Experimentally, a moderate increase—approximately twofold—is observed between the VCD signals recorded in a DMSO solution and in the solid state, a trend that is well reproduced by calculations.^56,95^ In the 3 μm region discussed here, the VCD signal arises entirely from non-local effects. Therefore, instead of describing this as an enhancement (since no signal is present for the isolated molecule), we refer to it as the *genesis* of the signal through non-local effects.

## Conclusions

It has been demonstrated that solid-state VCD in the 3 μm region provides detailed insights into the three-dimensional structure of molecular crystals, as exemplified by two chiral alcohols. In this region, the hydrogen-bonded network is probed by the strongly delocalised *ν*(OH) vibration. Due to the stable geometry found in the crystal, the relative orientation of molecules is preserved according to the space group symmetry, which leads to strong non-local VCD bands. A link between these VCD terms and the symmetry relations between molecules can be established by means of AIMD simulations and periodic NVPT calculations. While IR absorption is primarily observed within the hydrogen-bonded network, even weak vibrational coupling between molecules lacking hydrogen bonds can result in significant VCD signals. This demonstrates that these signals are not dependent on the presence of strong non-covalent interactions. VCD can thus be considered “symmetry-determined”, in contrast to the vDOS and IR absorption, which are “interaction-determined”. However, the appealing simplicity of such labels should not obscure the fact that it is the non-covalent interactions that maintain the geometry and orientation of the molecules in the crystal, which is essential for the generation of non-local VCD.

For the *ν*(OH) vibration, in contrast to the fingerprint region, no local chiroptical signal based on molecular chirality can be identified, although in all cases the OH groups are directly attached to the molecular chiral centres. It can thus be concluded that, for the systems studied here, any VCD measured experimentally in the 3 μm region has its origin in the crystal chirality, defined by the way the molecules are arranged in three-dimensional space. This exclusivity permits chiroptical studies of supramolecular effects without the interference of local molecular chirality. It is similarly anticipated that this generalises to any achiral molecule crystallising in Sohncke space groups.

Non-local contributions can be thought of as through-space coupling of local dipole moments carried by delocalised vibrations. Whereas IR absorption requires the alignment of two electric dipole vectors, VCD requires the alignment of one magnetic and one electric dipole moment. One might inquire as to why non-local coupling for IR absorption should be less susceptible to supramolecular geometry than VCD. The main reason is that in fact not one but two non-local terms, direct coupling (DC) and gauge transport (GT), contribute to VCD which find their individual maxima at different geometric conditions (see ref. 56 for details). This allows VCD to cover a wide geometric range, extending further into space.

Moreover, the fact, that the sign of the rotational strength depends on the relative orientation of the electric and magnetic dipole moments, allows for a much more detailed resolution of the *ν*(OH) band. This is particularly the case for the geometrically more complex space groups such as the *P*3_1_21 shown here, due to the superposition of both the DC and GT terms. Nevertheless, our findings demonstrate that for compound 1 with *P*2_1_ symmetry, non-local terms can also influence IR absorption: The distinctive shape, characterised by a double maximum, is accurately reproduced only when non-local effects are taken into account.

For the shown examples, AIMD simulations can be used to simulate vibrational spectra in the 3 μm region, although they do not take into account the quantum nature of the hydrogen atoms. In combination with periodic NVPT calculations, they yield spectra in very good agreement with experiment. They provide an accurate description of the long-range dynamical effects, which are essential since VCD is highly sensitive to weak vibrational coupling. This, however, can be a challenge when using classical force fields and neighbour lists.

It remains to be investigated whether the general concept that the full VCD spectrum can be reproduced from a small unit cell can be extended to biological soft matter such as DNA or protein fibrils, where long-range helical arrangements also play an important role.^43,96–99^ Furthermore, a comprehensive experimental-computational study of VCD to address molecular polymorphism has yet to be undertaken.

## Data availability

Data for this article, including raw data and detailed implementations are available at Zenodo at https://doi.org/10.5281/zenodo.7228747 (compound 1), https://doi.org/10.5281/zenodo.4776906 (compound 2), and https://doi.org/10.5281/zenodo.14222396. Further results and equations supporting this article have been included as part of the ESI.† The source codes of the development versions of ChirPy and CPMD are publicly available *via* the author's GitHub page https://github.com/sjaehnigen.

## Author contributions

SJ: data curation (theory), formal analysis, investigation, methodology (theory), resources, validation, visualization, writing – original draft, writing – review & editing, conceptualization, software, funding acquisition; RV: methodology (theory), writing – review & editing, funding acquisition; AZ: data curation (experiment), formal analysis, investigation, methodology (experiment), resources, writing – original draft, writing – review & editing, conceptualization, project administration, funding acquisition.

## Conflicts of interest

There are no conflicts to declare.

## Supplementary Material

SC-016-D4SC08055F-s001
