# Supplementary material for: The genesis of OH-stretching vibrational circular dichroism in chiral molecular crystals
Source: Chem Sci. 2025 Apr 22;16(22):9833–42. doi: 10.1039/d4sc08055f (PMC12044347; doi:10.1039/d4sc08055f)
Supplement: SC-016-D4SC08055F-s001 [file SC-016-D4SC08055F-s001.pdf]

Supplementary Information (SI) for Chemical Science.
This journal is © The Royal Society of Chemistry 2025

Supporting Information: The genesis of OH-stretching vibrational circular dichroism in chiral molecular crystals

Sascha Jähnigen,*,† Rodolphe Vuilleumier,‡ and Anne Zehnacker*,¶

†*Department of Biology, Chemistry, Pharmacy, Freie Universität Berlin, 14195 Berlin, Germany*

‡*PASTEUR, Département de Chimie, Ecole Normale Supérieure, PSL University, Sorbonne Université, CNRS, 75005 Paris, France*

¶*Institut des Sciences Moléculaires d'Orsay (ISMO), CNRS, Université Paris-Saclay, 91405 Orsay, France*

E-mail: sascha.jaehnigen@fu-berlin.de; anne.zehnacker-rentien@universite-paris-saclay.fr

Further details of the experimental procedure

The central frequency of the photo-elastic modulator (PEM) was set to 2500 cm^{-1}. Special care has been taken to prepare an isotropic sample so that the Rosenfeld equation is valid. To this end, KBr was first ground in a mixer mill (MM 400 Retsch) for 30 min at 20 Hz and kept dry in an oven at 80°C. Then 1g of KBr was mixed in the same conditions with 5 mg of the molecule under study. Approx. 150 mg of the resulting mixture was pressed as a transparent pellet and used as it was. Several pellets were made for each sample, among which only the non-scattering ones were kept. The linear dichroism of the selected pellets was tested as follows: the PEM was used in the half-wave mode and the lock-in detection was set at the second harmonic of the PEM frequency. The linear dichroism signal measured thereby was checked, and only the most promising samples were used for the VCD measurements. A continuously rotating homemade mount was first used for rotating

the sample during the VCD measurement, with a rotation speed adjustable between 1 and 20 Hz. However, it turned out that optimising the orientation of the pellet to minimise linear dichroism and applying the artefacts correction procedure proposed by Merten and derived from that introduced by Buffeteau gave more reliable results,[S1,S2] so it is the procedure that we chose. Each VCD spectrum corresponds to a 4 h measurement. The spectra of at least two pellets was recorded to check the reproducibility of the results.

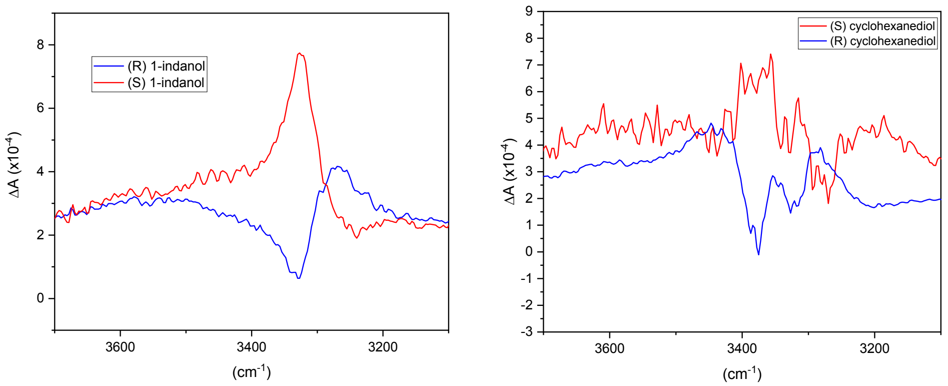

Figure S1: Experimental solid-state VCD spectra of the two enatiomers of compounds **1** (left) and **2** (right) obtained from a KBr pellet.

Theory and further results

As stated in the main text, due to the gauge dependence of the magnetic dipole moment, the non-local VCD term is split into a direct coupling (DC) and an origin-dependent gauge transport (GT) term,[S3,S4]

$$\langle \mathbf{j}_k(0) \cdot \mathbf{m}_l(\tau) \rangle = \underbrace{\langle \mathbf{j}_k \cdot \tilde{\mathbf{m}}_l \rangle}_{\text{DC}} + \underbrace{\frac{1}{2c} \langle \mathbf{j}_k \cdot (\mathrm{r}_l \times \mathbf{j}_l) \rangle}_{\text{GT}}, \tag{S1}$$

where $\tilde{\mathbf{m}}_l$ denotes the local magnetic dipole moment of unit l.

Under periodic boundary conditions, required for models of molecular crystals, the GT term takes a slightly different form to avoid spurious terms due to superfluous lattice translations,[S3]

$$\Delta\varepsilon(\omega) \propto \langle \mathbf{j}(0) \cdot \mathbf{m}(t) \rangle + \frac{1}{2c} \sum_i \left\langle \mathbf{j}_i(0) \cdot \sum_j \left[\left(\mathbf{r}_j(t) + \frac{1}{2} \Delta_{ij}^{\text{PBC}}(0) \right) \times \mathbf{j}_j(t) \right] \right\rangle, \tag{S2}$$

where Δ_{ij}^{PBC} is a conditional lattice translation in order to meet the nearest image convention between units i and j.

The local and non-local expressions for the vibrational density of states are

$$\left\langle \dot{q}(0) \cdot \dot{q}(\tau) \right\rangle = \underbrace{\sum_k \left\langle \dot{q}_k(0) \cdot \dot{q}_k(\tau) \right\rangle}_{\text{local}} + \underbrace{\sum_k \sum_{l \neq k} \left\langle \dot{q}_k(0) \cdot \dot{q}_l(\tau) \right\rangle}_{\text{non-local}}, \tag{S3}$$

where $\dot{q}_k(t)$ denotes the time derivative of the vibrational mode $q_k(t)$ of unit k (in this work, the two ν(OH) vibrations per unit are considered with $|\dot{q}_k| = |\dot{q}_{k,1}| + |\dot{q}_{k,2}|$).

The local and non-local expressions for the IR absorption spectrum are

$$\left\langle \mathbf{j}(0) \cdot \mathbf{j}(\tau) \right\rangle = \underbrace{\sum_k \left\langle \mathbf{j}_k(0) \cdot \mathbf{j}_k(\tau) \right\rangle}_{\text{local}} + \underbrace{\sum_k \sum_{l \neq k} \left\langle \mathbf{j}_k(0) \cdot \mathbf{j}_l(\tau) \right\rangle}_{\text{non-local}}. \tag{S4}$$

Only DC terms terms arise for vDOS or IR absorption.

Crystal symmetry in (S, S)-1,2-*trans*-cyclohexanediol

Table S1: Symmetry operations of the P$3_1$21 space group.

Symmetry operation A	Jones faithful notation		
1	x	y	z
3_1	$-y$	$x-y$	$\frac{1}{3}+z$
$3_2 \equiv 3_{-1}$	$-x+y$	$-x$	$\frac{2}{3}+z$
2	y	x	$-z$
$2 \oplus 3_1$	$x-y$	$-y$	$\frac{2}{3}-z$
$2 \ominus 3_1$	$-x$	$-x+y$	$\frac{1}{3}-z$

A symmetry-adapted non-local term can be derived by identifying and extracting from the double sum those pair couplings that belong to a specific symmetry class of the space group (NB: 3_1 and 3_2 belong to the same symmetry class),

$$\begin{aligned} \left\langle \mathbf{j}(0) \cdot \mathbf{m}(\tau) \right\rangle &= \left\langle \sum_k \mathbf{j}_k(0) \cdot \sum_l \mathbf{m}_l(\tau) \right\rangle \\ &= \underbrace{\sum_k \left\langle \mathbf{j}_k(0) \cdot \mathbf{m}_k(\tau) \right\rangle}_{1\equiv\text{local}} + \underbrace{\sum_{k,l}^{3_1/3_2} \left\langle \mathbf{j}_k(0) \cdot \mathbf{m}_l(\tau) \right\rangle + \sum_{k,l}^{2} \left\langle \mathbf{j}_k(0) \cdot \mathbf{m}_l(\tau) \right\rangle}_{\text{non-local}} \\ &\quad + \underbrace{\sum_{k,l}^{2\oplus 3_1} \left\langle \mathbf{j}_k(0) \cdot \mathbf{m}_l(\tau) \right\rangle + \sum_{k,l}^{2\oplus 3_2} \left\langle \mathbf{j}_k(0) \cdot \mathbf{m}_l(\tau) \right\rangle}_{\text{non-local}} \end{aligned} \tag{S5}$$

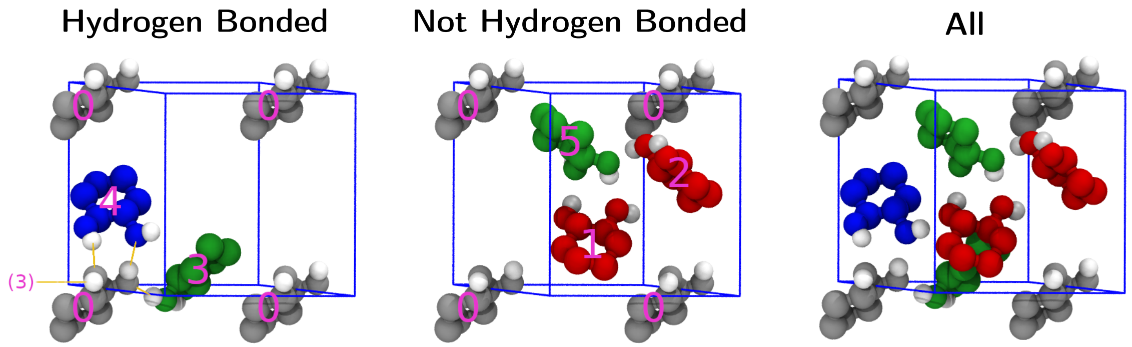

Figure S2: Location of molecular (asymmetric) units in the unit cell of compound **2**. For clarity, the molecule of reference (0) is shown as four periodic images. The colours correspond to the symmetry relations used in Figure 6.

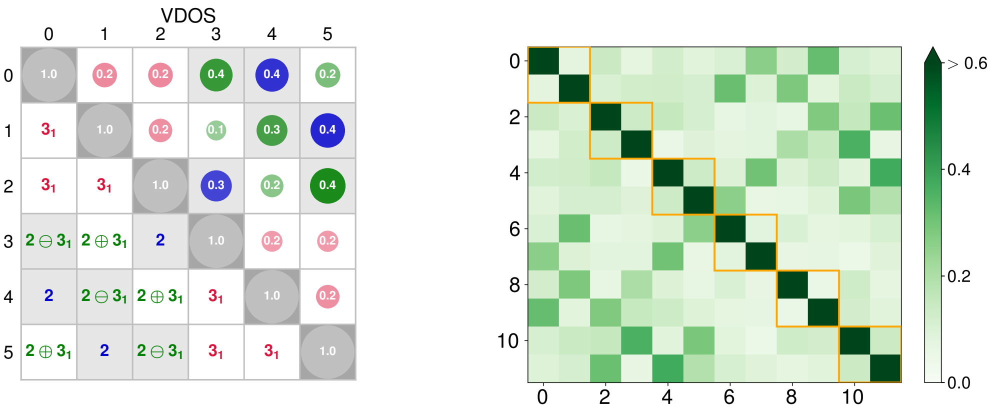

Figure S3: Left: Symmetry relations and correlation matrix of the vDOS of the six molecules (labelled 0 to 5) found in the unit cell of $(S,\,S)$-1,2-*trans*-cyclohexanediol. The diagonal axis represents the local term; grey shaded off-diagonal cells indicate molecules connected by a hydrogen bond. Right: Correlation matrix of the vDOS, resolved for the twelve OH groups found in the unit cell. The yellow squares mark the OH pair of each molecule.

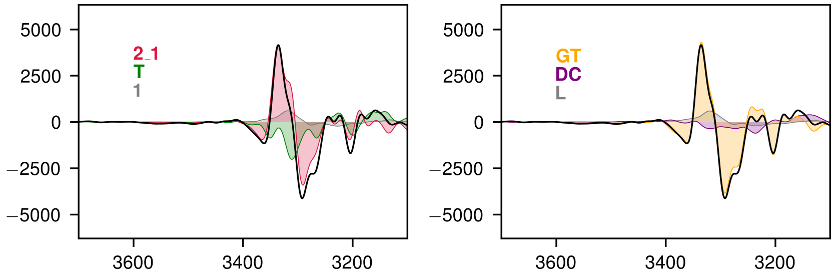

Figure S4: VCD spectral decomposition in local and non-local subterms of compound **1**. Left: contributions per symmetry operation (T: translation); right: contributions from local as well as non-local DC and GT terms (*cf.* Equation S1).

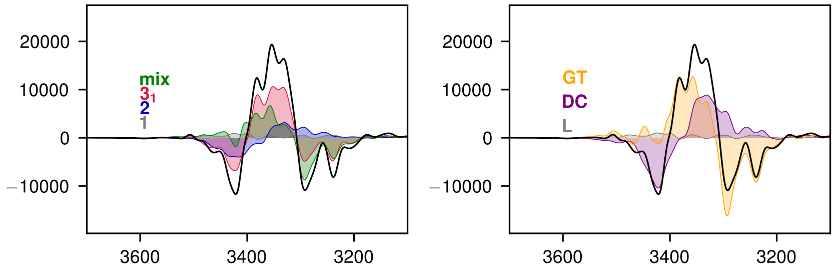

Figure S5: VCD spectral decomposition in local and non-local subterms of compound **2**. Left: contributions per symmetry operation; right: contributions from local as well as non-local DC and GT terms (*cf.* Equation S1).

References

(S1) Merten, C.; Kowalik, T.; Hartwig, A. Vibrational Circular Dichroism Spectroscopy of Solid Polymer Films: Effects of Sample Orientation. *Appl Spectrosc* **2008**, *62*, 901–905.

(S2) Buffeteau, T.; Lagugné-Labarthet, F.; Sourisseau, C. Vibrational Circular Dichroism in General Anisotropic Thin Solid Films: Measurement and Theoretical Approach. *Appl. Spectrosc., AS* **2005**, *59*, 732–745.

(S3) Jähnigen, S.; Zehnacker, A.; Vuilleumier, R. Computation of Solid-State Vibrational Circular Dichroism in the Periodic Gauge. *J. Phys. Chem. Lett.* **2021**, *12*, 7213–7220.

(S4) Jähnigen, S.; Le Barbu-Debus, K.; Guillot, R.; Vuilleumier, R.; Zehnacker, A. How Crystal Symmetry Dictates Non-Local Vibrational Circular Dichroism in the Solid State. *Angew. Chem. Int. Ed.* **2023**, *62*, e202215599.
